# HEAVY DRINKING, MENTAL HEALTH PROBLEMS AND RECOVERY IN OLDER ADULTS: EXPLORING SUPPORT NEEDS

**DOI:** 10.1093/geroni/igad104.3388

**Published:** 2023-12-21

**Authors:** Deepti John, Bethany Bareham, Amy O’Donnell, Jennifer Liddle, Carolyn Chew-Graham, Eileen Kaner, Barbara Hanratty

**Affiliations:** Newcastle University, Newcastle upon Tyne, England, United Kingdom; Newcastle University, Newcastle upon Tyne, England, United Kingdom; Newcastle University, Newcastle upon Tyne, England, United Kingdom; Newcastle University, Newcastle upon Tyne, England, United Kingdom; Keele University, Keele, England, United Kingdom; Newcastle University, Newcastle upon Tyne, England, United Kingdom; Newcastle University, Newcastle upon Tyne, England, United Kingdom

## Abstract

Many older adults who consume alcohol at harmful levels experience mental health problems. Common life events such as retirement, bereavement, and declining health, can contribute to anxiety and depression, with alcohol consumption being used as a coping mechanism. Tailored support is crucial to address the unique needs of older adults facing both alcohol misuse and mental health problems. These patients often fall into gaps between services (family practice/alcohol/mental health), which are unable to meet their complex support needs. This qualitative study aimed to understand the lived experiences of older adults with co-occurring heavy drinking and mental health problems, support received and unmet needs. We conducted semi-structured interviews with 14 older adults living in the North of England (completed Summer 2023). Thematic analysis and constant comparison were employed in data analysis. Findings highlighted the importance of age-tailored initiatives such as access to mental health support while actively drinking, supervised detoxification, increased peer support and services that are age inclusive and friendly. Risk factors such as grief, loneliness, financial problems and undiagnosed trauma in older adults contributed to development of these conditions. Older adults need services to work together, integrated around patient needs and addressing heavy drinking and mental health concerns at the same time. Future research should focus on improving mental health literacy and, developing holistic and preventive interventions to address risk factors for co-occurring alcohol and mental health problems in older adults.

